# Three new species of *Hamataliwa* Keyserling, 1887 from Xizang, China (Araneae, Oxyopidae)

**DOI:** 10.3897/zookeys.1282.187584

**Published:** 2026-06-23

**Authors:** Chaojun Long, Shilin Wang, Jincan Zhang, Cheng Wang, Xiaoqi Mi

**Affiliations:** 1 Guizhou Provincial Key Laboratory for Biodiversity Conservation and Utilization in the Fanjing Mountain Region, Tongren University, Tongren 554300, Guizhou, China College of Health Sciences, Kaili University Guizhou China; 2 College of Health Sciences, Kaili University, Kaili 556000, Guizhou, China Guizhou Provincial Key Laboratory for Biodiversity Conservation and Utilization in the Fanjing Mountain Region, Tongren University Guizhou China

**Keywords:** DNA barcodes, lynx spider, morphology, Qomolangma, taxonomy

## Abstract

Three new species of *Hamataliwa* Keyserling, 1887, collected from Xizang, China, are described: *H. jinlini* Wang & Mi, **sp. nov**. (♂♀), *H. qomolangma* Wang & Mi, **sp. nov**. (♂♀), and *H. shufui* Wang & Mi, **sp. nov**. (♂♀). The dissected bulb of the genus is shown for the first time. Diagnostic photos of the habitus and copulatory organs, as well as a map of the collecting localities of the type specimens for the new species, are provided. DNA barcodes of all new species were obtained for future reference.

## Introduction

*Hamataliwa* Keyserling, 1887, the second-largest genus within the family, currently contains 91 extant species widely distributed in North and South America, Sub-Saharan Africa, Australia, and East, South and Southeast Asia (WSC 2026). The genus is relatively well studied, with revisions available for North and Central America, Borneo, and China (Brady 1964, 1970; Zhang et al. 2005; Deeleman-Reinhold 2009; Tang and Li 2012). However, the taxonomic study of the genus remains unsatisfactory: a quarter of its species lack diagnostic drawings or photos, preventing accurate identification, and one-sixth are known only from a single sex or even from subadults (WSC 2026).

Up to now, 18 species have been recorded in China, of which 17 are endemic (WSC 2026). Xizang covers more than 1.2 million square kilometers and lies southwest of the Qinghai-Xizang Plateau, which has long been known as the roof of the world (Zhou et al. 2025). To date, only six species of *Oxyopes* Latreille, 1804 have been recorded in this autonomous region (WSC 2026). In the past three years, our field surveys have revealed the presence of *Hamataliwa* in Xizang, and three new species were recognized as new after comparison with other congeners. The present work aims to describe them.

## Material and methods

Specimens were collected by beating shrubs or hand collecting. They were preserved in 90% ethanol. Specimens are deposited in the museum of Tongren University (TRU) in Tongren, China. They were examined with an Olympus SZX 16 stereomicroscope. After dissection, the epigynes were cleared in trypsin enzyme solution and imaged in ethanol and lactic acid, as well as arabic gum. Left male palps were used for the descriptions and illustrated in ethanol. Photographs of the copulatory organs and habitus were taken with a Kuy Nice CCD camera mounted on an Olympus BX43 compound microscope. Focus-stacked images were generated using Helicon Focus v. 6.7.1. ArcGIS v. 10.8 software was used to create a distribution map.

DNA barcodes were obtained for three new species for future reference. A partial fragment of the mitochondrial cytochrome *c* oxidase subunit I (COI) gene was amplified and sequenced using the primers COI-TY-F1 and COI-TY-R1 (Yamasaki et al. 2018). The accession numbers are provided in Table 1.

**Table 1.** Voucher specimen information.

Species	Voucher code	Sex	GenBank accession number
*Hamataliwa jinlini*	TRU-Oxy-0002	♂	PZ239916
*Hamataliwa jinlini*	TRU-Oxy-0004	♀	PZ239917
*Hamataliwa jinlini*	TRU-Oxy-0005	♀	PZ239918
*Hamataliwa qomolangma*	TRU-Oxy-0018	♂	PZ239919
*Hamataliwa qomolangma*	TRU-Oxy-0024	♀	PZ239920
*Hamataliwa shufui*	TRU-Oxy-0013	♂	PZ239921
*Hamataliwa shufui*	TRU-Oxy-0014	♀	PZ239922

All measurements are given in millimeters. Leg measurements are given as total length (femur, patella, tibia, metatarsus, tarsus). Leg spination follows Amulya et al. (2022) and d, p, v, r for dorsal, prolateral, ventral and retrolateral sides of the segments. Abbreviations used in the text and figures are as follows: **ALE** anterior lateral eye; **AME** anterior median eye; **aRTA** anterior ramus of retrolateral tibial apophysis; **At** atrium; **C** conductor; **CD** copulatory duct; **CF** cymbial fold; **CO** copulatory opening; **E** embolus; **FD** fertilization duct; **H** epigynal hood; **MA** median apophysis; **NNR** National Nature Reserve; **PA** prolateral apophysis; **PL** posterior lobe; **PLE** posterior lateral eye; **PME** posterior median eye; **pRTA** posterior ramus of retrolateral tibial apophysis; **RPA** retrolateral patellar apophysis; **RTA** retrolateral tibial apophysis; **S** spermatheca; **SE** sclerotized edge; **Sp** septum; **VTA** ventral tibial apophysis.

## Taxonomy

### Family Oxyopidae Thorell, 1879

#### Genus Hamataliwa

Taxon classificationAnimaliaAraneaeOxyopidae

Keyserling, 1887

CD3775D1-7E41-5B69-84D8-6F383BA5C25F

Hamataliwa Keyserling, 1887: 457.
Emmenophrys Simon, 1898: 33 (synonymized by Simon 1903 with Hamataliwa). Type species: E. porcatus Simon, 1898 from Brazil.
Oxyopeidon O. Pickard-Cambridge, 1894: 139 (synonymized by Bryant 1948 with Hamataliwa). Type species: O. difficile O. Pickard-Cambridge, 1894 from Mexico.
Megullia Thorell, 1897: 30 (synonymized by Deeleman-Reinhold 2009 with Hamataliwa). Type species: M. truncata Thorell, 1897 from Vietnam.
Hamataliwa : Lo et al. 2024: 18.

##### Type species.

*Hamataliwa grisea* Keyserling, 1887 from North America.

##### Distribution.

North and South America, Sub-Saharan Africa, Australia, and East, South and Southeast Asia (WSC 2026).

#### Hamataliwa jinlini

Taxon classificationAnimaliaAraneaeOxyopidae

Wang & Mi sp. nov.

01FC5A6C-C0F5-5515-86FA-988C87DA8EFD

https://zoobank.org/578C5846-FFF4-4F9D-BB75-D23AFA94AF92

**Figure 1.**
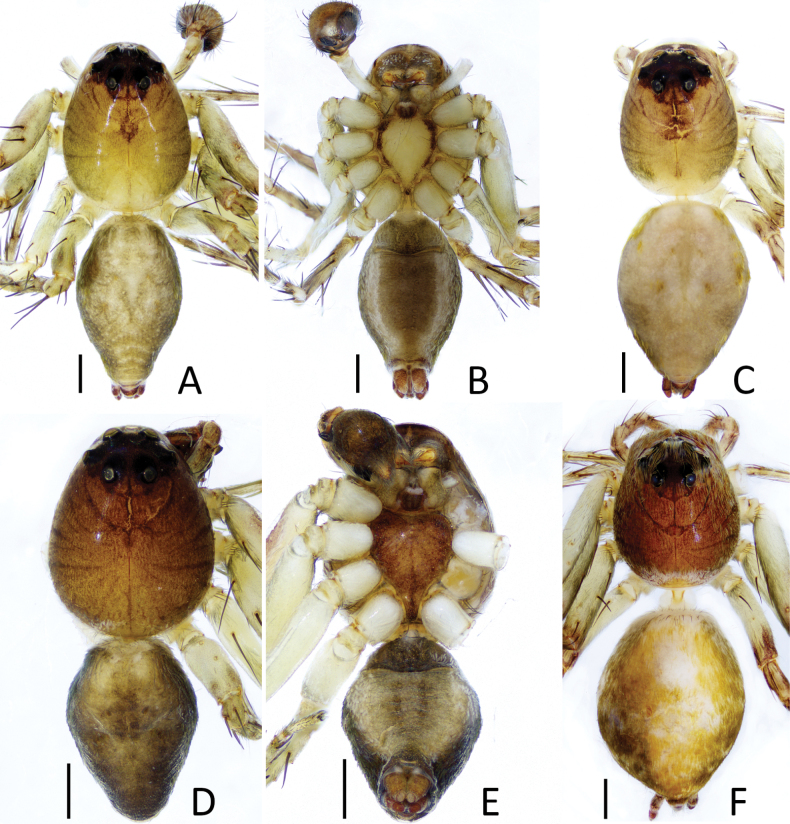
Habitus of *Hamataliwa* spp. **A**–**C**. *H. jinlini* Wang & Mi, sp. nov., **D**–**F**. *H. shufui* Wang & Mi, sp. nov. **A, B, D, E**. Male holotype; **C, F**. Female paratypes (**C**. TRU-Oxy-0004; **F**. TRU-Oxy-0014). **A, C, D, F**. Dorsal view; **B, E**. Ventral view. Scale bars: 0.5 mm.

Figs 1A–C, 3, 6A, 6B, 7, 10

##### Type material.

***Holotype***: China • ♂ (TRU-Oxy-0001); Xizang Auton. Region, Linzhi City, Bomi Co., Gu Twp., Qiaona Vill., Yarlung Zangbu Grand Canyon NNR (30°1.96'N, 95°14.12'E, ca 2450 m), 20 May 2024, X.Q. Mi et al. leg. ***Paratypes***: • 2♂3♀ (TRU-Oxy-0002–0006), same data as for holotype; • 1♀ (TRU-Oxy-0007), Motuo Co., Paimo Hwy, Yarlung Zangbu Grand Canyon NNR (29°28.10'N, 94°59.44'E, ca 3210 m), 23 May 2024, X.Q. Mi et al. leg.; • 2♀ (TRU-Oxy-0008–0009), Motuo Co., Renqingbengsi, Yarlung Zangbu Grand Canyon NNR (29°18.31'N, 95°21.29'E, ca 1970 m), 26 May 2024, X.Q. Mi et al. leg.; • 2♀ (TRU-Oxy-0010–0011), Motuo Co., Damu Twp., Zhu Vill., Yarlung Zangbu Grand Canyon NNR (29°29.73'N, 95°25.86'E, ca 1740 m), 27 May 2024, X.Q. Mi et al. leg.

##### Etymology.

The specific name is after the late Prof. Jinlin Hu (胡金林), who made significant contributions to the taxonomic study of spiders of the Qinghai-Xizang Plateau.

##### Diagnosis.

The male of the new species is similar to that of *H. wangi* Lin & Li, 2022 in the shape of the embolus (E) and conductor (C), but can be easily distinguished by the following: (1) median apophysis (MA) is divided into two sheet-shaped lamellas distally in ventral view (Figs 3A, 6A) vs hook-shaped (Lin et al. 2022: fig. 30A); and (2) the tip of the ventral tibial apophysis (VTA) is directed towards about 3:00 o’clock position in ventral view (Fig. 3A) vs about 1:00 (Lin et al. 2022: fig. 30A). The female of this new species is similar to that of *H. pedicula* Tang & Li, 2012 in the shape of the copulatory ducts (CD) and spermathecae (S), but can be easily distinguished by the copulatory ducts, which originate from the median portion of the epigyne (Fig. 7B, D) vs from posterior portion of the epigyne (Tang and Li 2012: figs 13C, 14B) and by the posterior sclerotized edge (SE), which is almost arc-shaped (Fig. 7A, C) vs just slightly curved (Tang and Li 2012: figs 13B, 14A).

##### Description.

**Male** (holotype, Figs 1A, 1B, 3; paratype, TRU-Oxy-0002, Fig. 6A, B). Total length 4.60. Carapace 2.23 long, 1.67 wide. Abdomen 2.30 long, 1.40 wide. Eye sizes and interdistances: AME 0.13, ALE 0.23, PME 0.20, PLE 0.17, AME–AME 0.10, AME–ALE 0.12, ALE–PLE 0.33, PME–PLE 0.37, PME–PME 0.35. Legs: I 7.71 (2.10, 0.68, 2.05, 1.90, 0.98); II 7.18 (2.02, 0.65, 1.93, 1.75, 0.83); III 5.70 (1.70, 0.55, 1.30, 1.45, 0.70); IV 5.40 (1.48, 0.53, 1.23, 1.48, 0.68). Carapace elliptical, yellow-brown with brown stripes; eye region dark. Chelicerae dark brown in general, anterior surface with dense pale scales. Endites yellow, covered with short setae. Labium mainly dark, with dense, anteromarginal dark setae. Sternum yellow. Legs pale yellow, spination of leg I: femur d2 p3 r2; patella d1 p1; tibia d2 p4 r4; metatarsus d3 p4 r4. Abdomen long, elliptical, dorsum yellow-brown, with inconspicuous pattern; venter darker than dorsum, with broad, dark brown median band extending from epigastric furrow to hind end.

**Figure 2.**
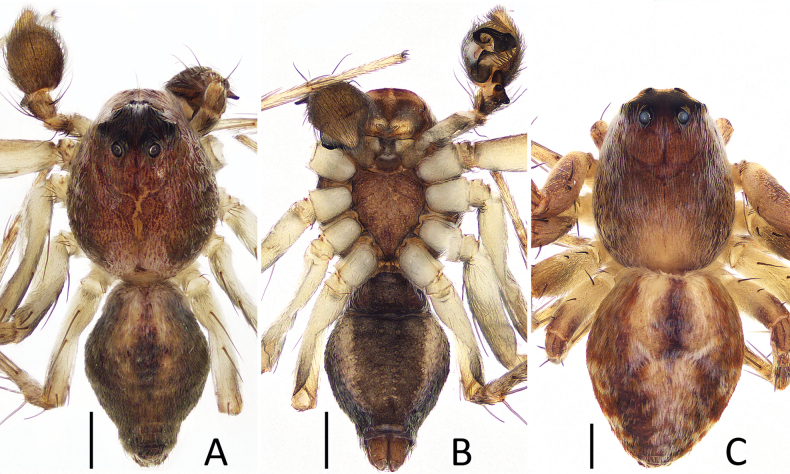
Habitus of *Hamataliwa qomolangma* Wang & Mi, sp. nov. **A, B**. Male holotype; **C**. Female paratype, TRU-Oxy-0024; **A, C**. Dorsal view; **B**. Ventral view. Scale bars: 0.5 mm.

**Figure 3.**
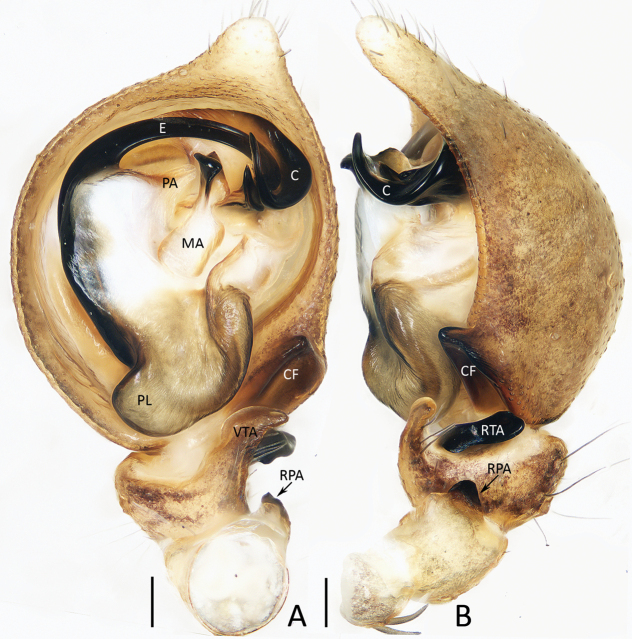
Male palp of *Hamataliwa jinlini* Wang & Mi, sp. nov., holotype. **A**. Ventral view; **B**. Retrolateral view. Abbreviations: C conductor; CF cymbial fold; E embolus; MA median apophysis; PA prolateral apophysis; PL posterior lobe; RPA retrolateral patellar apophysis; RTA retrolateral tibial apophysis; VTA ventral tibial apophysis. Scale bars: 0.1 mm.

***Palp*** (Figs 3, 6A, B): femur length/width ratio about 2.2; patella with sub-triangular antero-retrolateral apophysis (RPA) in retrolateral view; ventral tibial apophysis (VTA) weakly sclerotized, curved towards retrolateral side and with blunt tip directed towards about 3:00 o’clock position in ventral view; retrolateral tibial apophysis (RTA) strongly sclerotized, sheet-shaped in retrolateral view; cymbium length/width ratio ca 1.4, with baso-retrolateral fold (CF); tegulum with postero-prolaterally extended posterior lobe (PL); median apophysis (MA) narrowest medially, and divided into 2 sheet-shaped lamellas distally, with sub-square base in ventral view; embolus (E) curved about half circle, tip hidden by conductor; conductor (C) anteroretrolaterally located, strongly sclerotized, tip curved to half circle.

**Female** (paratype, TRU-Oxy-0004, Figs 1C, 7). Total length 4.34. Carapace 1.88 long, 1.43 wide. Abdomen 2.25 long, 1.66 wide. Eye sizes: AME 0.11, ALE 0.19, PME 0.16, PLE 0.19. Eye interdistances: AME–AME 0.13, AME–ALE 0.12, ALE–PLE 0.41, PME–PLE 0.36, PME–PME 0.41. Legs: I missing; II 5.66 (1.67, 0.46, 1.49, 1.29, 0.75); III 4.49 (1.35, 0.39, 1.06, 1.07, 0.62); IV 4.49 (1.34, 0.44, 0.96, 1.13, 0.62). Habitus (Fig. 1C) similar to that of male except paler in color. Spination of leg I: missing.

***Epigyne*** (Fig. 7): almost as long as wide, with medio-posterior atrium (At) and posterior, arc-shaped sclerotized edge (SE); copulatory openings (CO) beneath anterolateral portion of atrium, opened posteriorly; copulatory ducts (CD) curved to inverted C-shape; spermathecae (S) globular, about 3/4 their diameters apart.

##### Distribution.

China (Xizang, Fig. 10).

#### Hamataliwa qomolangma

Taxon classificationAnimaliaAraneaeOxyopidae

Wang & Mi sp. nov.

D951C96C-503C-53F9-9FF9-DE178D209836

https://zoobank.org/BC8F66BE-6656-4355-A576-21717C6B24C7

**Figure 4.**
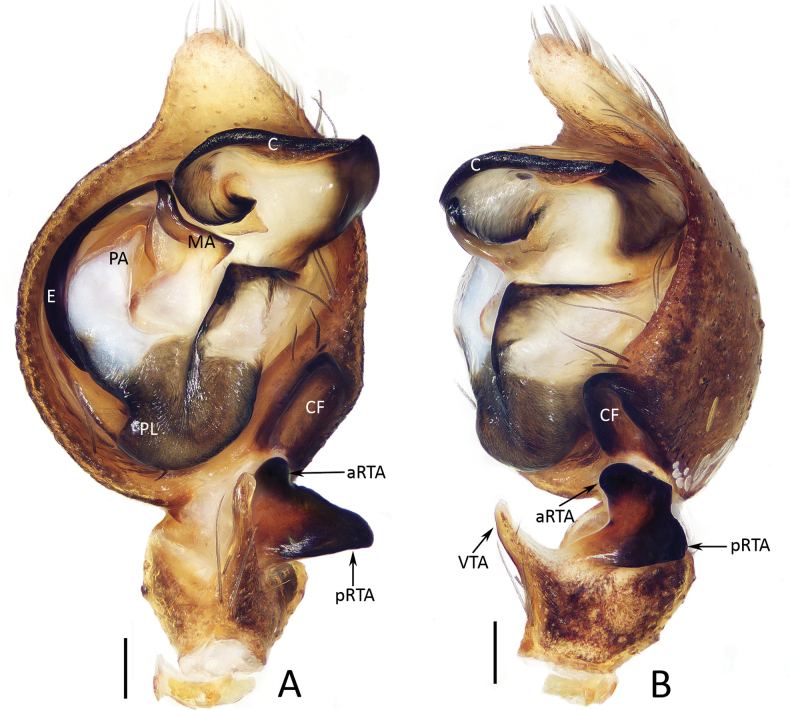
Male palp of *Hamataliwa qomolangma* Wang & Mi, sp. nov., holotype. **A**. Ventral view; **B**. Retrolateral view. Abbreviations: aRTA anterior ramus of retrolateral tibial apophysis; C conductor; CF cymbial fold; E embolus; MA median apophysis; PA prolateral apophysis; PL posterior lobe; pRTA posterior ramus of retrolateral tibial apophysis; VTA ventral tibial apophysis. Scale bars: 0.1 mm.

Figs 2, 4, 6 C, D, 8, 10

##### Type material.

***Holotype***: China • ♂ (TRU-Oxy-0017); Xizang Auton. Region, Rikaze City, Dingjie Co., Chentang Twp., Natang Vill., Qomolangma NNR (27°50.28'N, 87°26.28'E, ca 2390 m), 27 May 2025, C. Wang et al. leg. ***Paratypes***: • 6♂8♀ (TRU-Oxy-0018–0031), same data as for holotype; • 2♀ (TRU-Oxy-0032–0033), Dingjie Co., Chentang Twp., Chentanggou, Qomolangma NNR (27°51.78'N, 87°25.65'E, ca 2370 m), 18 August 2024, C. Wang et al. leg.; • 1♂1♀ (TRU-Oxy-0034–0035), Jilong Co., Sare Twp., Labi Vill. Qomolangma NNR (28°18.99'N, 85°24.99'E, ca 2530 m), 25 May 2025, X.Q. Mi et al. leg.

##### Etymology.

The specific epithet is a noun in apposition referring to the Qomolangma National Nature Reserve, the type locality.

##### Diagnosis.

The new species is similar to *H. cucullata* Tang, Wang & Peng, 2012 in the general shape of copulatory organs, especially the form of ventral tibial apophysis (VTA), epigynal hood (H), copulatory ducts (CD) and spermathecae (S), but can be easily distinguished by the following: (1) ventral tibial apophysis (VTA) is about half the tibial length in retrolateral view (Fig. 4B) vs about as long as tibia (Tang et al. 2012: figs 6, 13); (2) the posterior ramus of the retrolateral tibial apophysis (pRTA) is directed towards dorsal side in retrolateral view (Fig. 4B) vs retrolateral side (Tang et al. 2012: figs 6, 13); (3) epigynal hood (H) is partly extending beyond the base of septum and is wider than septum (Fig. 8A, C, D) vs not extending beyond the base of septum and about 2/3 the septum width (Tang et al. 2012: figs 8, 10, 14); and (4) atrium (At) is almost trapezoidal (Fig. 8) vs almost oval (Tang et al. 2012: figs 8, 10, 14).

##### Description.

**Male** (holotype, Figs 2A, 2B, 4; paratype, TRU-Oxy-0018, Fig. 6C, D). Total length 3.41. Carapace 1.79 long, 1.38 wide. Abdomen 1.62 long, 1.15 wide. Eye sizes and interdistances: AME 0.06, ALE 0.12, PME 0.09, PLE 0.18, AME–AME 0.09, AME–ALE 0.06, ALE–PLE 0.24, PME–PLE 0.24, PME–PME 0.21. Legs: I 5.55 (1.49, 0.53, 1.47, 1.33, 0.73); II 5.02 (1.50, 0.48, 1.33, 1.28, 0.43); III 4.11 (1.29, 0.23, 1.03, 0.93, 0.63); IV 3.91 (1.05, 0.39, 0.90, 1.02, 0.55). Carapace oval, red-brown, with median, longitudinal, yellow stripe, eye region dark, margin covered with dense grey setae. Chelicerae dark brown in general. Endites yellow, covered with short setae. Labium dark brown, with pale anterior edges. Sternum red-brown. Legs slender, pale yellow, spination of leg I: femur d1 p3; patella d1 r1; tibia d2 p4 r4; metatarsi d2 p5 r5. Abdomen long, elliptical, dorsum dark brown, setose, with center, longitudinal, fusiform dark patch; venter darker than dorsum, with broad, dark brown band extending across whole surface, covered with short setae.

***Palp*** (Figs 4, 6C, D) femur length/width ratio ca 1.4; patella almost as long as wide; ventral tibial apophysis (VTA) about half of tibial length, slightly curved and with blunt end in retrolateral view; retrolateral tibial apophysis (RTA) with half round anterior ramus (aRTA) and sub-triangular posterior ramus (pRTA) in ventral view; cymbium length/width ratio ca 1.4, with baso-retrolateral fold (CF); tegulum with prolaterally extended posterior lobe (PL); median apophysis (MA) postero-prolateral to conductor (C), divided into dorsal sheet-shaped ramus and near bar-shaped ventral ramus curved slightly; embolus (E) tapered, curved clockwise, distally covered by conductor; conductor (C) almost transversely extended, with anterior curly margin, hollowed medially.

**Female** (paratypes, TRU-Oxy-0024, Figs 2C, 8A, 8B, and TRU-Oxy-0025, Fig. 8C, D). Total length 4.21. Carapace 2.04 long, 1.57 wide. Abdomen 2.18 long, 1.75 wide. Eye sizes and interdistances: AME 0.08, ALE 0.11, PME 0.11, PLE 0.11, AME–AME 0.08, AME–ALE 0.06, ALE–PLE 0.19, PME–PLE 0.22, PME–PME 0.15. Legs: I 6.46 (1.86, 0.65, 1.73, 1.42, 0.80); II 6.17 (1.81, 0.63, 1.60, 1.33, 0.80); III 5.71 (1.68, 0.60, 1.62, 1.08, 0.73); IV 4.73 (1.40, 0.55, 1.08, 1.15, 0.55). Habitus (Fig. 2C) similar to that of male except paler in color. Spination of leg I: femur d3 p1 r2; patella d1 r1; tibia d2 p4 r4; metatarsi d2 p5 r5.

***Epigyne*** (Fig. 8): ~ 1.2× longer than wide, with oval, posterior hood (H) hollowed centrally; atrium (At) almost trapezoidal, with longitudinal septum (Sp) about 2/5 atrium length; copulatory openings (CO) open posterolaterally; copulatory ducts (CD) short, curved about 90° medially; spermathecae (S) almost globular.

##### Distribution.

China (Xizang, Fig. 10).

#### Hamataliwa shufui

Taxon classificationAnimaliaAraneaeOxyopidae

Wang & Mi sp. nov.

EC9FE98B-DF62-5AC6-BAC0-AAD30302C77D

https://zoobank.org/DC22FE5A-9740-48E0-90BA-B9BE69CED81B

**Figure 5.**
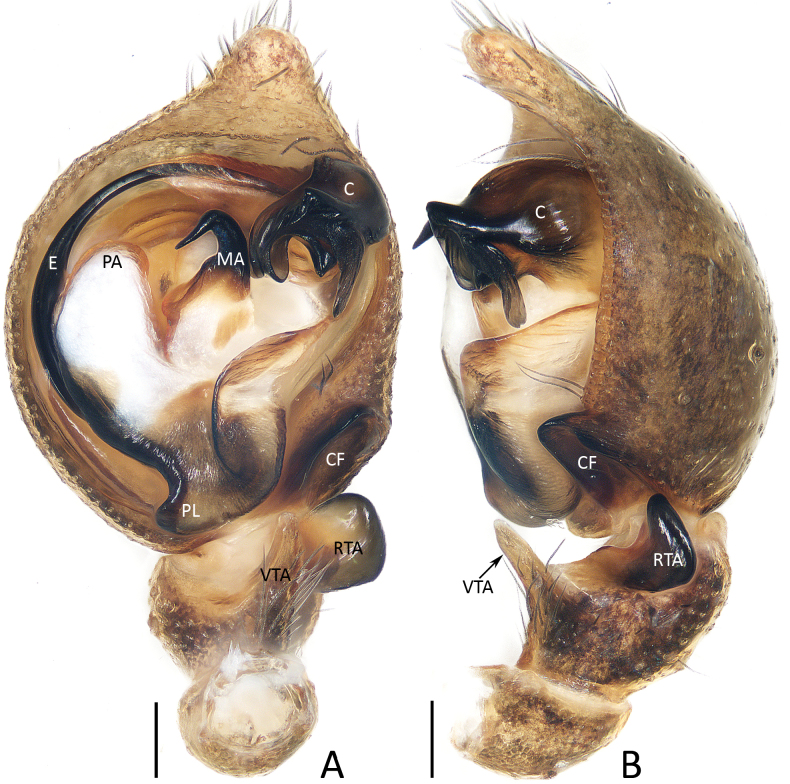
Male palp of *Hamataliwa shufui* Wang & Mi, sp. nov., holotype. **A**. Ventral view; **B**. Retrolateral view. Abbreviations: C conductor; CF cymbial fold; E embolus; MA median apophysis; PA prolateral apophysis; PL posterior lobe; RTA retrolateral tibial apophysis; VTA ventral tibial apophysis. Scale bars: 0.1 mm.

**Figure 6.**
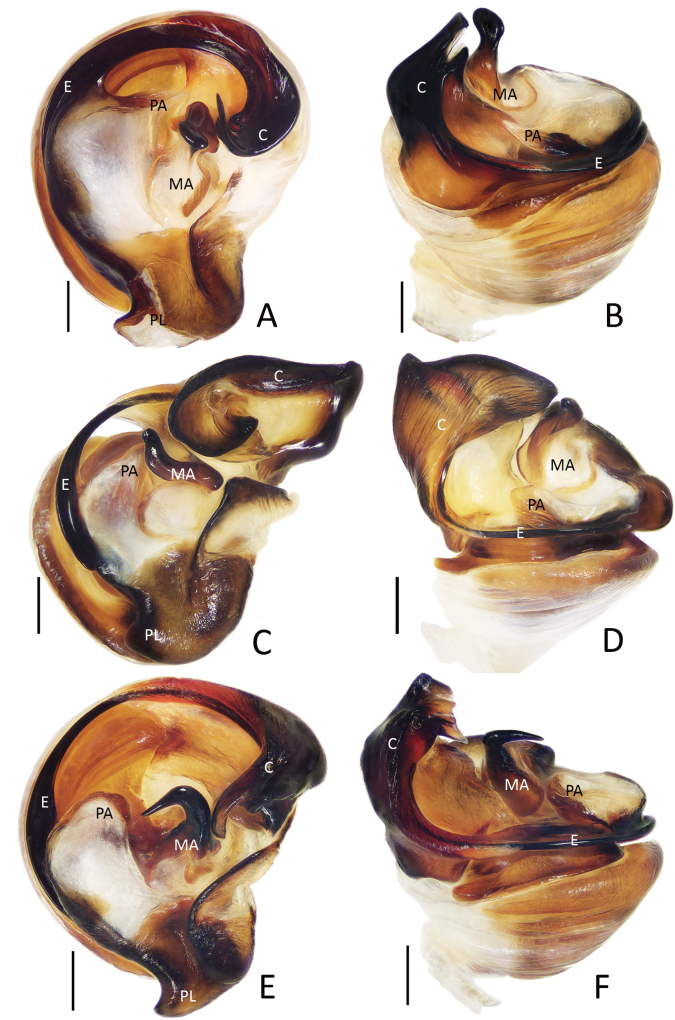
Bulb of *Hamataliwa* spp. **A, B**. *H. jinlini* Wang & Mi, sp. nov., paratype, TRU-Oxy-0002; **C, D**. *H. qomolangma* Wang & Mi, sp. nov., paratype, TRU-Oxy-0018; **E, F**. *H. shufui* Wang & Mi, sp. nov., paratype, TRU-Oxy-0013. **A, C, E**. Ventral view; **B, D, F**. Anterior view. Abbreviations: C conductor; E embolus; MA median apophysis; PA prolateral apophysis; PL posterior lobe. Scale bars: 0.1 mm.

Figs 1D–F, 5, 6E, 6F, 9, 10

##### Type material.

***Holotype***: China • ♂ (TRU-Oxy-0012); Xizang Auton. Region, Linzhi City, Motuo Co., Damu Twp., Zhu Vill., Yarlung Zangbu Grand Canyon NNR (29°29.73'N, 95°25.86'E, ca 1740 m), 27 May 2024, X.Q. Mi et al. leg. ***Paratypes***: • 1♂ (TRU-Oxy-0013), same data as for holotype; • 1♀ (TRU-Oxy-0014), Bomi Co., Gu Twp., Qiaona Vill., Yarlung Zangbu Grand Canyon NNR (30°1.96'N, 95°14.12'E, ca 2450 m), 20 May 2024, X.Q. Mi et al. leg.; • 1♂1♀ (TRU-Oxy-0015–0016), Motuo Co., Renqingbengsi, Yarlung Zangbu Grand Canyon NNR (29°18.31'N, 95°21.29'E, ca 1970 m), 26 May 2024, X.Q. Mi et al. leg.

##### Etymology.

The specific name is in honor of Fu Shu (舒服) (Changsha, China), for his assistance in collecting specimens in the Yarlung Zangbu Grand Canyon.

##### Diagnosis.

The male of the new species is similar to that of *H. cordata* Zhang, Zhu & Song, 2005 in having a hook-shaped median apophysis (MA) and a ramose conductor (C), but can be easily distinguished by the following: (1) ventral tibial apophysis (VTA) tapers in ventral view (Fig. 5A) vs broadened distally (Zhang et al. 2005: fig. 9); and (2) prolateral apophysis (PA) extends anteriorly in ventral view (Fig. 5A) vs anteroretrolaterally (Zhang et al. 2005: fig. 9). The female of this new species is similar to that of *H. cucullata* Tang, Wang & Peng, 2012 in having an epigynal hood (H), but can be distinguished by the following: (1) copulatory openings (CO) open anteriorly (Fig. 9A, C) vs posterolaterally (Tang et al. 2012: figs 8, 10, 14); (2) atrium (At) is approximately 1.75× wider than long (Fig. 9A, C) vs approximately 1.2× wider than long (Tang et al. 2012: figs 8, 10, 14); and (3) epigynal hood (H) is hollowed centrally (Fig. 9A, C) vs not hollowed (Tang et al. 2012: figs 8, 10, 14).

**Figure 7.**
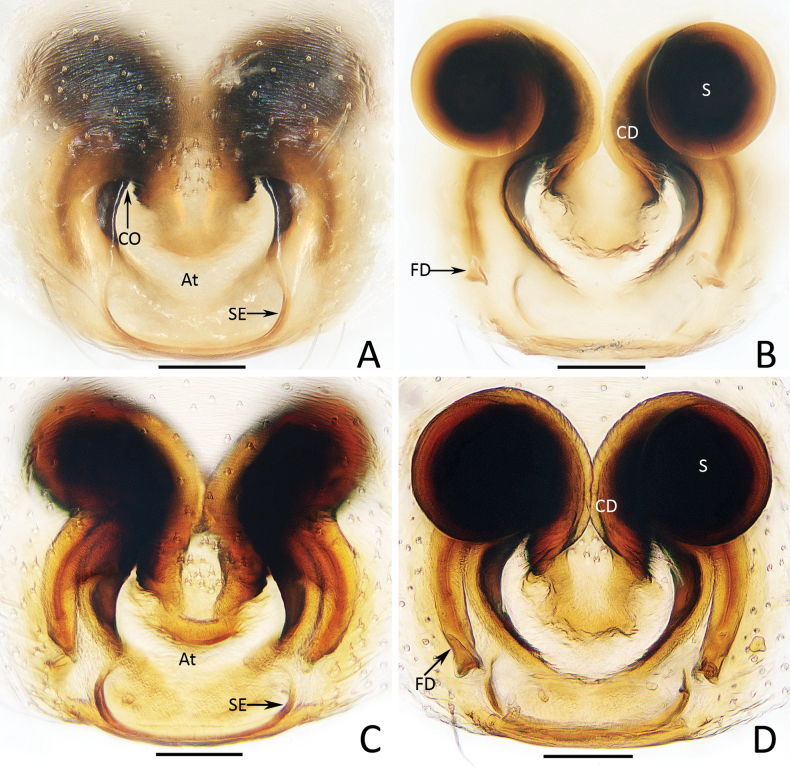
Epigyne of *H. jinlini* Wang & Mi, sp. nov., paratype, TRU-Oxy-0004. **A, C**. Ventral view; **B, D**. Dorsal view. **A**. In ethanol; **B**. In lactic acid; **C, D**. In arabic gum. Abbreviations: At atrium; CO copulatory opening; CD copulatory duct; FD fertilization duct; S spermatheca; SE sclerotized edge. Scale bars: 0.1 mm.

**Figure 8.**
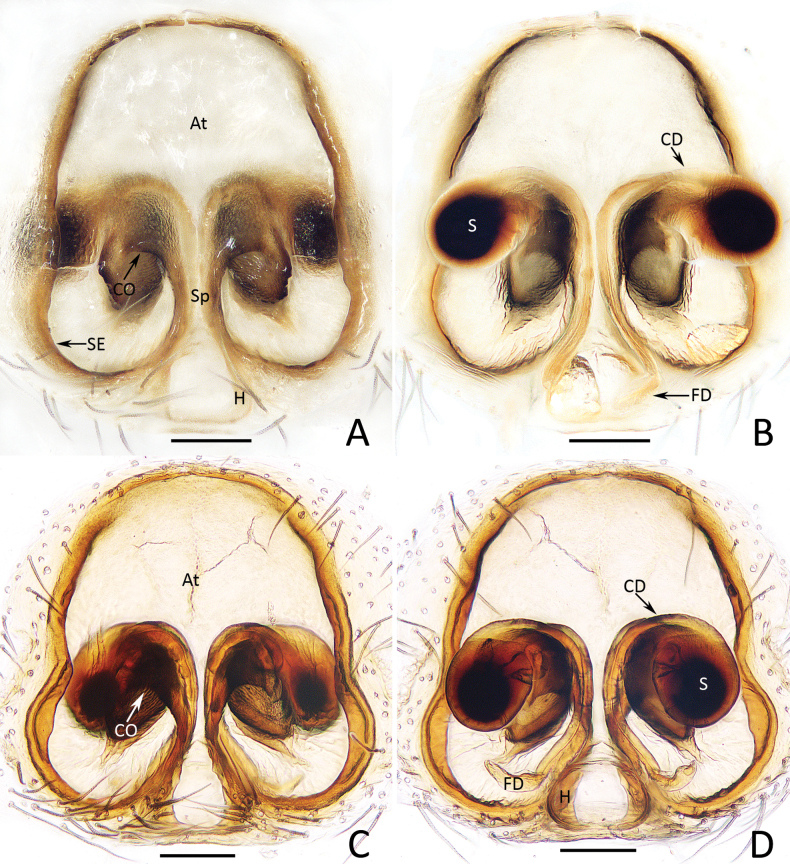
Epigyne of *Hamataliwa qomolangma* Wang & Mi, sp. nov., paratypes (**A, B**. TRU-Oxy-0024; **C, D**. TRU-Oxy-0025). **A, C**. Ventral view; **B, D**. Dorsal view. **A**. In ethanol; **B**. In lactic acid; **C, D**. In arabic gum. Abbreviations: At atrium; CO copulatory opening; CD copulatory duct; FD fertilization duct; H epigynal hood; S spermatheca; SE sclerotized edge; Sp septum. Scale bars: 0.1 mm.

##### Description.

**Male** (holotype, Figs 1D, 1E, 5; paratype, TRU-Oxy-0013, Fig. 6E, F). Total length 3.54. Carapace 1.89 long, 1.48 wide. Abdomen 1.64 long, 1.18 wide. Eye sizes and interdistances AME 0.09, ALE 0.17, PME 0.13, PLE 0.16; AME–AME 0.09, AME–ALE 0.06, ALE–PLE 0.19, PME–PLE 0.27, PME–PME 0.19. Legs: I 6.02 (1.66, 0.53, 1.57, 1.41, 0.85); II 5.57 (1.52, 0.42, 1.50, 1.37, 0.76); III 4.46 (1.37, 0.44, 1.02, 1.06, 0.57); IV 4.07 (1.09, 0.36, 0.87, 1.13, 0.62). Carapace oval, red-brown with irregular yellow stripe behind eye area and lateral dark brown stripes, posteriorly covered with white scales, eye area dark. Chelicerae red-brown. Endites yellow, covered with short setae. Labium mainly brown, with dense, anteromarginal dark setae. Sternum red-brown. Legs pale yellow, spination of leg I: femur d2 p2 r1; patella d1 r1; tibia d1 p4 r3; metatarsi d1 p3 r4. Abdomen long-elliptical, dorsum yellow-brown, darker posteriorly, with median, faint patches; venter pale yellow in general, covered with short setae.

***Palp*** (Figs 5, 6E, F): femur length/width ratio ca 1.74; patella almost as long as wide; ventral tibial apophysis (VTA) straight, tapered to blunt end directed towards about 12:00 o’clock position in ventral view; retrolateral tibial apophysis (RTA) sub-square in ventral view; cymbium length/width ratio ca 1.3, with baso-retrolateral fold (CF); tegulum with anteriorly extended prolateral apophysis (PA) and postero-prolaterally extended posterior lobe; median apophysis (MA) enlarged and weakly sclerotized at base, remainder hook-shaped and with pointed end; embolus (E) curved to half circle and distally hidden by conductor (C); conductor anteroretrolaterally located, ramose distally.

**Figure 9.**
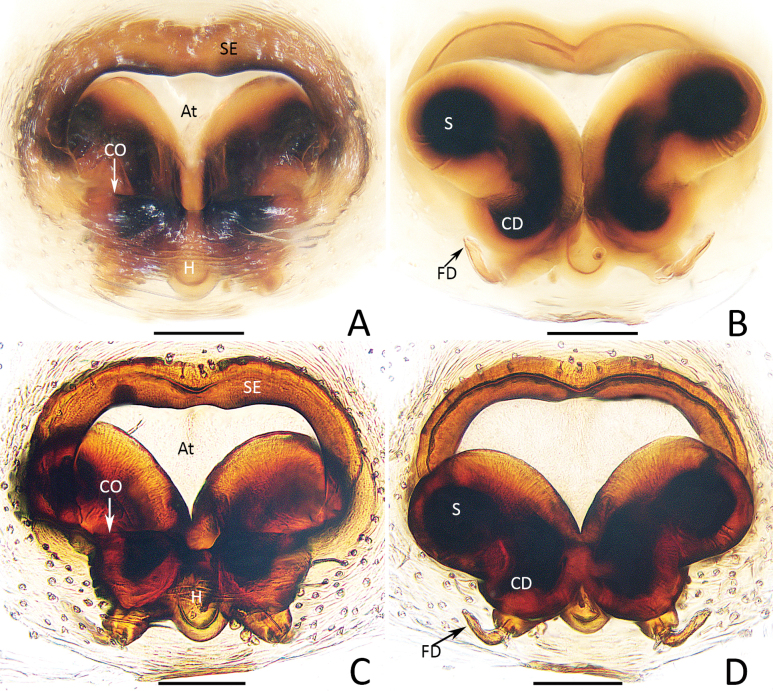
Epigyne of *Hamataliwa shufui* Wang & Mi, sp. nov., paratype, TRU-Oxy-0014. **A, C**. Ventral view; **B, D**. Dorsal view. **A**. In ethanol; **B**. In lactic acid; **C, D**. In arabic gum. Abbreviations: At atrium; CO copulatory opening; CD copulatory duct; FD fertilization duct; H epigynal hood; S spermatheca; SE sclerotized edge. Scale bars: 0.1 mm.

**Female** (paratype, TRU-Oxy-0014, Figs 1F, 9). Total length 4.77. Carapace 2.27 long, 1.65 wide. Abdomen 2.50 long, 1.92 wide. Eye sizes and interdistances: AME 0.10, ALE 0.17, PME 0.13, PLE 0.13; AME–AME 0.13, AME–ALE 0.08, ALE–PLE 0.19, PME–PLE 0.25, PME–PME 0.21. Legs: I 6.13 (1.85, 0.56, 1.59, 1.36, 0.77); II 5.44 (1.60, 0.35, 1.49, 1.21, 0.79); III 4.80 (1.46, 0.43, 1.12, 1.16, 0.63); IV 4.57 (1.21, 0.49, 1.03, 1.18, 0.66). Habitus (Fig. 1F) similar to that of male, except carapace smaller in size and abdomen paler in color. Spination of leg I: femur d3 p1 r2; patella d1 p1; tibia d2 p4 r4; metatarsi d2 p5 r5.

***Epigyne*** (Fig. 9): ~ 1.4× wider than long, with central, sub-oval atrium (At), and posterior, bell-shaped hood (H) open anteriorly; copulatory openings (CO) anterolateral to hood; copulatory ducts (CD) thick, curved to about half circle; spermathecae (S) without distinct border, almost oval, touching each other.

##### Distribution.

**Figure 10.**
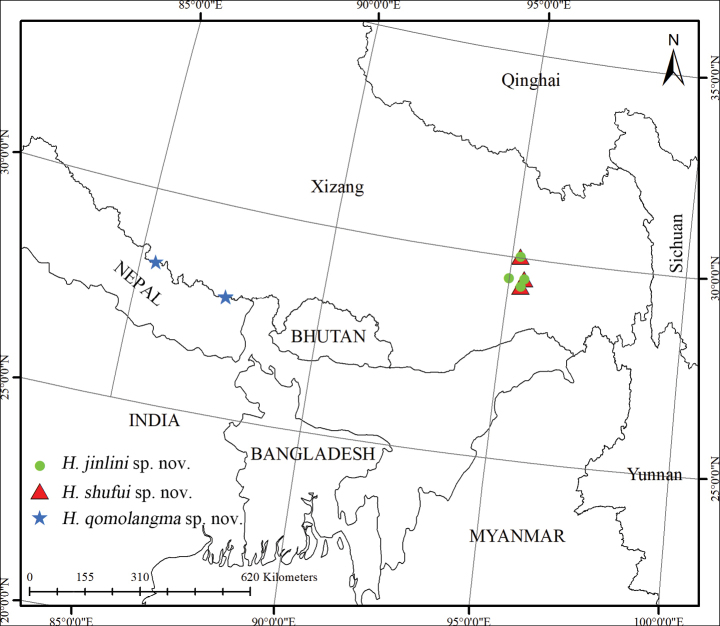
Map of collecting localities of the type specimens of the three *Hamataliwa* species.

China (Xizang, Fig. 10).

## Supplementary Material

XML Treatment for Hamataliwa

XML Treatment for Hamataliwa jinlini

XML Treatment for Hamataliwa qomolangma

XML Treatment for Hamataliwa shufui

## Additional information

### Artificial Intelligence (AI) use

The authors accept full responsibility for the content of the manuscript, including the disclosure of any use of AI.

No AI tools were used in the preparation of this manuscript.

### Author ORCIDs

C. Long https://orcid.org/0009-0005-6577-7341

S. Wang https://orcid.org/0009-0003-0759-5350

J. Zhang https://orcid.org/0009-0002-9726-4485

C. Wang https://orcid.org/0000-0003-1831-0579

X. Mi https://orcid.org/0000-0003-1744-3855

### Data availability

All of the data that support the findings of this study are available in the main text.
